# Ursolic Acid Ameliorates Inflammation in Cerebral Ischemia and Reperfusion Injury Possibly *via* High Mobility Group Box 1/Toll-Like Receptor 4/NFκB Pathway

**DOI:** 10.3389/fneur.2018.00253

**Published:** 2018-05-18

**Authors:** Yanzhe Wang, Lei Li, Shumin Deng, Fang Liu, Zhiyi He

**Affiliations:** Department of Neurology, The First Affiliated Hospital of China Medical University, Shenyang, China

**Keywords:** brain ischemia, ursolic acid, toll-like receptors, inflammatory mediators, high mobility group box 1

## Abstract

Toll-like receptors (TLRs) play key roles in cerebral ischemia and reperfusion injury by inducing the production of inflammatory mediators, such as interleukins (ILs) and tumor necrosis factor-alpha (TNF-α). According to recent studies, ursolic acid (UA) regulates TLR signaling and exhibits notable anti-inflammatory properties. In the present study, we explored the mechanism by which UA regulates inflammation in the rat middle cerebral artery occlusion and reperfusion (MCAO/R) model. The MCAO/R model was induced in male Sprague–Dawley rats (MCAO for 2 h, followed by reperfusion for 48 h). UA was administered intragastrically at 0.5, 24, and 47 h after reperfusion. The direct high mobility group box 1 (HMGB1) inhibitor glycyrrhizin (GL) was injected intravenously after 0.5 h of ischemia as a positive control. The degree of brain damage was estimated using the neurological deficit score, infarct volume, histopathological changes, and neuronal apoptosis. We assessed IL-1β, TNF-α, and IL-6 levels to evaluate post-ischemic inflammation. HMGB1 and TLR4 expression and phosphorylation of nuclear factor kappa-light-chain-enhancer of activated B cell (NFκB) were also examined to explore the underlying mechanism. UA (10 and 20 mg/kg) treatment significantly decreased the neurological deficit scores, infarct volume, apoptotic cells, and IL-1β, TNF-α, and IL-6 concentrations. The infarct area ratio was reduced by (33.07 ± 1.74), (27.05 ± 1.13), (27.49 ± 1.87), and (39.74 ± 2.14)% in the 10 and 20 mg/kg UA, GL, and control groups, respectively. Furthermore, UA (10 and 20 mg/kg) treatment significantly decreased HMGB1 release and the TLR4 level and inactivated NFκB signaling. Thus, the effects of intragastric administration of 20 mg/kg of UA and 10 mg/kg of GL were similar. We provide novel evidence that UA reduces inflammatory cytokine production to protect the brain from cerebral ischemia and reperfusion injury possibly through the HMGB1/TLR4/NFκB signaling pathway.

## Introduction and Background

Ischemic stroke, which occurs as a result of the sudden occlusion of a blood vessel by a thrombus or embolism, is a common cause of death and disability worldwide (1). Currently, thrombolysis therapy within the therapeutic window and mechanical thrombectomy in stroke patients are widely accepted for the treatment of sudden cerebral ischemia (2, 3). However, an inflammatory response has been shown to occur after thrombolysis, exacerbating the reperfusion injury (4–6). Therefore, studies aiming to identify an effective adjunct to treatments for cerebral ischemia and reperfusion injury deserve more attention.

Toll-like receptor 4 (TLR4) plays a key role in cerebral ischemia and reperfusion injury by inducing the production of inflammatory mediators, such as interleukins (ILs) and tumor necrosis factor-alpha (TNF-α) (7, 8). TLR4 were initially identified as receptors for endogenous ligands known as damage-associated molecular patterns (DAMPs), particularly high mobility group box 1 (HMGB1), during brain injury. HMGB1 is a ubiquitous DNA-binding nuclear protein that is either passively released from necrotic cells or actively secreted in response to inflammatory signals (9, 10). In addition, overactive microglia and reactive astrocytes in the ischemic region can aggravate ischemic damage after activation of the TLR4 signaling pathways (11). Therefore, strategies that modulate post-ischemic TLR4 signaling in the brain may suppress inflammation induced by cerebral ischemia and provide new therapies for stroke.

Ursolic acid (UA: 3b-hydroxy-urs-12-ene-28-oic acid), a natural pentacyclic triterpenoid, has been reported to exhibit biological activities in the brain, including anti-oxidative, anti-tumor, anti-rheumatic, anti-viral, and anti-inflammatory effects (12). Furthermore, UA also inhibited microglial and astrocyte activation and decreased the levels of TNF-α, IL-1β, and IL-6 in lipopolysaccharide-induced brain inflammation in mice with cognitive deficits (13). However, researchers have not determined whether UA protects against ischemia and reperfusion injury by antagonizing the HMGB1/TLR4 signaling pathway. In this study, we used glycyrrhizin (GL) as a positive control drug. GL is a direct HMGB1 inhibitor and the effective dose for treating cerebral ischemia and reperfusion injury has been established (14).

In the present study, we used the rat middle cerebral artery occlusion and reperfusion (MCAO/R) model with UA and GL to examine the mechanism by which UA regulates the inflammation response induced by ischemia and reperfusion. We investigated whether UA reduced inflammatory cytokine production to protect the brain from cerebral ischemia and reperfusion injury possibly though the HMGB1/TLR4/NFκB signaling pathway.

## Materials and Methods

### Animal Preparation and Drug Administration

All experimental protocols involving animals were performed according to the guidelines of the National Institutes of Health Guide for the Care and Use of Laboratory Animals (Publication No. 85-23, revised 1985), the UK Animals Scientific Procedures Act 1986 or the European Communities Council Directive of 24 November 1986 (86/609/EEC) and the “Guiding Principles in the Use of Animals in Toxicology,” adopted by the Society of Toxicology in 1989. All procedures used in animal experiments were approved by the Institutional Animal Care and Use Committee of China Medical University. Ninety male Sprague–Dawley rats were purchased from the Liaoning Changsheng Biotechnology Company (Benxi, China). These rats were housed under a 12-h light/12-h day cycle with free access to food and water *ad libitum*. Rats weighing between 250 and 280 g were randomly divided into five groups. (1) In the sham group (*n* = 18), rats underwent the same surgical procedures as rats in the MCAO/R group without filament insertion and received the vehicle. (2) In the control group (*n* = 18), rats underwent the MCAO/R surgical procedures and received vehicles both intragastrically (i.g.) and intravenously (i.v.) when the other treatment groups were administered UA or GL. (3) In the low-dose UA (L-UA) group (*n* = 18), 10 mg/kg UA (purity ≥ 95.0%, Sigma-Aldrich, St. Louis, MO, USA) in distilled water containing 0.5% Tween-80 (ddH_2_O/0.5% Tween-80) was administered by oral gavage at 0.5, 24, and 47 h after reperfusion, according to previous studies clarifying the oral absorption rate and drug action time (15–17). (4) The high-dose UA (H-UA) group (*n* = 18) was administered 20 mg/kg UA. (5) The GL group rats (*n* = 18) were i.v. administered 10 mg/kg of GL (purity ≥ 95.0%, Sigma-Aldrich, St. Louis, MO, USA) in a volume of 0.5 ml of distilled water containing 0.5% Tween-80 (ddH_2_O/0.5% Tween-80) *via* the tail vein 0.5 h after ischemia and before reperfusion as a positive control (18–21).

According to previous studies clarifying the oral absorption rate and drug action time, UA-administered mice had a lethal dose 50 of 60 mg/kg and a rat-to-mouse dosing ratio of 6.3/9.1. The final dose of UA was 5, 10, and 20 mg/kg. Since UA is insoluble in water, 0.1% Tween-80 is used as a solubilizer and 0.1% Tween-80 is used to dilute UA to 1 mg/ml, and the pH is adjusted to 7.4 to avoid the acid and alkali caused by the drug stimulate.

Forty-eight hours after reperfusion, 18 rats in each group were randomly divided into three groups by a researcher who was unaware of the neurological deficits in these rats. Six rats were decapitated to obtain fresh brain tissue samples for biochemical analyses. The ischemic cortex, which was defined as the penumbra, was collected for ELISA and western blotting analyses based on methods modified from Jiang et al. (22). The brains of six rats were stained to determine the infarct volume; six rats were perfused with fixative for histological preparation and analysis of the brains. The brain samples from each animal were sectioned into three slices beginning 3 mm from the anterior tip of the frontal lobe in the coronal plane. The slices were 3-, 4-, and 3-mm thick from front to back, respectively. The middle slices were embedded in paraffin and sliced into 5-μm thick sections for Nissl staining, immunohistochemical staining, immunofluorescence staining, and double-labeling using terminal deoxynucleotidyl transferase-mediated dUTP nick end labeling (TUNEL) and neuronal nuclei (NeuN). To ensure that the positive cells were counted at the same coronal level, we collected ten 5-μm thick coronal sections of the dorsal hippocampus (−3.3 to −4.5 mm from the bregma). The number of positive cells in each section was averaged from three non-overlapping fields at the same site of the middle cerebral artery blood supply in the ischemic (right) cortex within the penumbral area based on methods modified from previous studies (23, 24).

The success rate of model preparation in this experiment was 83.3%. No neurological impairment was observed in 3 of them, 3 with score of 4, 2 died in surgery, 14 with subarachnoid hemorrhage. Subarachnoid hemorrhage accounted for 77.8% of excluded factors, as the main excluded factor.

### Experimental Transient Middle Cerebral Artery Occlusion Model

Surgical procedures for MCAO/R were performed in rats using the intracranial suture method, as previously described (25). Briefly, a 5-cm nylon monofilament (diameter, 0.26 mm) with a rounded tip coated with silicon (Guangzhou Jialing Biotechnology Company) was inserted into the right internal carotid artery to block the origin of the MCA (approximately 18 ± 2 mm) and maintained for 120 min. Rats in the sham group underwent the same surgical procedures without the insertion of a filament. The rectal temperature was maintained above 36.5°C during and after the surgery with a heating pad. Cerebral blood flow (CBF) was monitored throughout the entire operation. The success of the MCAO/R model was defined as a decrease in CBF by at least 80% during MCA occlusion and a return to 80% CBF after reperfusion.

### Analysis of Neurological Deficits

A five-point scale of neurologic deficit scores was used to evaluate neurological behavior. The neurological deficits were scored 48 h after reperfusion by other investigators who were blinded to the experimental groups (*n* = 18 in each group). The scoring criteria for neurological deficits have been described previously by Longa et al. (25) and Bederson et al. (26, 27).

### Infarct Volume Measurements

Infarct volume was assessed 48 h after reperfusion (*n* = 6 per group) with 2,3,5-triphenyltetrazolium chloride (TTC, Sigma), as previously described in detail (28, 29). The stained slices were photographed and quantified using ImagePro Plus 6.0. Lesion volumes were corrected using the following formula to compensate for the effect of post-ischemic edema on the volume of the injury (26, 30):

$$\begin{array}{l} \text{Percentage of corrected infarct volume}\\ \;=[\text{Contralateral hemisphere area}\\ \;-(\text{Ipsilateral hemisphere area }-\text{ Measured infarct area})]\\ \;/\text{ Contralateral hemisphere area}*100\%. \end{array}$$

### Nissl Staining

Sections were deparaffinized and then incubated with a 1% cresyl violet (Sigma) solution for Nissl staining. Images were captured using a light microscope (at 400× magnification). In the Nissl-stained sections, only intact neurons were counted.

### Double-Labeling Using TUNEL and NeuN

A TUNEL assay was performed according to the manufacturer’s instructions (Roche Molecular Biochemicals, Inc., Mannheim, Germany). Sections were incubated with rabbit anti-NeuN antibody (Cell Signaling Technology, Danvers, MA, USA) in PBS/0.2% TX-100 and then incubated with the TUNEL reaction mixture to verify the neuronal identity of the TUNEL-positive cells. Finally, 4′,6-diamidino-2-phenylindole (DAPI) was added. The total number of TUNEL-positive neurons was counted by an investigator who was blinded to the study protocol.

### Immunohistochemical Staining of HMGB1 and TLR4

Immunohistochemical staining of HMGB1 and TLR4 was performed using paraffin-embedded brain samples from each animal (*n* = 6 per group), which were sectioned and deparaffinized. The sections were incubated with an anti-HMGB1 monoclonal antibody (diluted 1:400, Cell Signaling Technology, Danvers, MA, USA) and an anti-TLR4 monoclonal antibody (diluted 1:100, Abcam PLC, Cambridge, UK). Binding was detected using the streptavidin-peroxidase kit (Maixin, Fuzhou, China). The positive cells were identified, counted, and analyzed in the sections with the ImageJ software.

### Immunofluorescence Staining of Iba-1 and GFAP

Immunofluorescence staining of the microglial marker Iba-1 and the astrocytic marker GFAP were performed using paraffin-embedded brain samples of rats (*n* = 6 per group) that had been sectioned and deparaffinized. Sections were incubated with primary antibodies (goat anti-Iba-1, 1:100, Abcam, Cambridge, UK, or rabbit anti-GFAP, 1:200, Abcam, Cambridge, UK) and then with secondary antibodies labeled with fluorescent dyes (rabbit anti-goat, 1:200, Santa Cruz Biotechnology, CA, USA, or mouse anti-rabbit, 1:200, Santa Cruz Biotechnology, CA, USA). Photomicrographs were quantified performed by converting the images to gray scale, inverting their color, and quantifying the staining intensity in each field with ImageJ software.

### Measurement of the IL-1β, TNF-α, IL-6, and Plasma HMGB1 Levels by ELISA

The IL-1β, IL-6, and TNF-α levels in the ischemic cortex and the HMGB1 levels in the plasma samples were determined using ELISA kits (USCN Life Science Inc., Wuhan, China) according to the manufacturer’s instructions.

### Isolation of Protein and Western Blot Analysis for HMGB1, TLR4, IκB, Phospho-IκB, NFκB p65, and Phospho-NFκB p65

Cytosolic and nuclear proteins from the ischemic cortex were prepared with the Nuclear/Cytosol Fractionation Kit (BioVision, Mountain View, CA, USA) for western blotting analysis. As previously described in detail, the protein samples were separated using 10% sodium dodecyl sulfate-polyacrylamide gel electrophoresis and then transferred to a polyvinylidene fluoride membrane (Millipore Corporation, Billerica, MA, USA). The membrane was incubated with the following antibodies: anti-HMGB1 antibody (diluted 1:1,000, Cell Signaling Technology), anti-TLR4 antibody (diluted 1:200, Abcam), anti-NFκB p65 antibody (diluted 1:500; Abcam), anti-IκB antibody (diluted 1:500, Abcam), anti-phospho-IκB antibody (diluted 1:500; Abcam), and phospho-NFκB p65 antibody (diluted 1:500; Abcam). To confirm equal loading, we used an anti-GAPDH antibody (1:500 dilution, Santa Cruz Biotechnology) and an anti-lamin A antibody (diluted 1:1,000, Abcam). The density of each band was quantified using ImageJ.

### Statistical Analysis

All data are expressed as mean ± SD and analyzed with one-way analysis of variance using SPSS20.0. *P* < 0.05 was defined as statistically significant. The neurological deficit scores among the different groups were compared using the Kruskal–Wallis test. When the Kruskal–Wallis test showed a significant difference, the Dunn’s multiple comparisons test was applied. Given the simple size of six animals per group, actual power was performed with the G*Power 3.1.9.2 software at 5% significance level. We got a power greater than 0.9.

## Results

### Effect of UA on Neurological Deficits in Rats With MCAO/R

**Figure 1:**
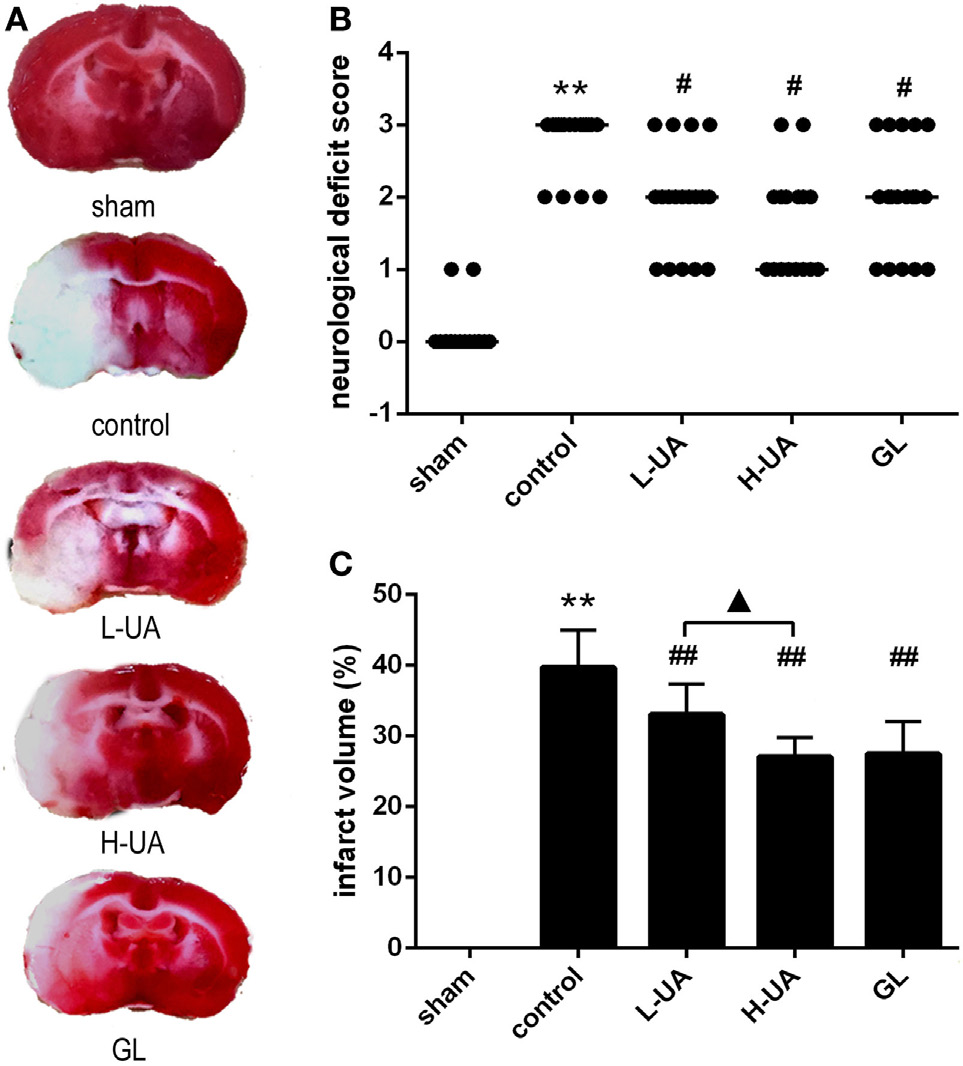
Effect of ursolic acid (UA) on neurological deficit scores and infarct volumes [values represent the mean ± SE, *n* = 18 (neurological deficit score) and *n* = 6 per group]. UA was administered intragastrically (i.g.) at 0.5, 24, and 47 h after reperfusion. Glycyrrhizin (GL), which is a direct high mobility group box 1 inhibitor, was injected intravenously (i.v.) after 0.5 h of ischemia. **(A)** Brain slices were stained with TTC. The red color indicates healthy tissue; white indicates infracted tissue. The fourth slice from the anterior region is presented (approximately −1 to −3 mm from the bregma) to show differences among the groups. **(B)** UA (10 and 20 mg/kg) and GL improved the neurological deficit scores (^#^*P*′ < 0.012). **(C)** UA (10 and 20 mg/kg) and GL reduced the infarct volume (^##^*P* < 0.01). The difference between the low-dose UA (L-UA) group and the high-dose UA (H-UA) group was significant (^▲^*P* < 0.05) (**P < 0.01: compared with the sham group; ^##^*P* < 0.01: compared with the control group; ^#^*P*′ < 0.012: compared with the control group; ^▲^*P* < 0.05 compared with the H-UA group).

After 48 h of reperfusion, neurological deficit scores were significantly increased in the control group (Figure 1B). The UA-treated group (10 and 20 mg/kg) and the GL-treated group displayed significant improvements in their general condition and in neurological deficits compared with the control group (Figure 1B). Moreover, rats treated with 20 mg/kg UA displayed lower median neurological deficit scores than rats treated with GL.

### Effect of UA on Infarct Volume in Rats With MCAO/R

The infarct volume was assessed 48 h after reperfusion by TTC staining. No obvious infarction could be observed in the sham group. The rats treated with UA (10 and 20 mg/kg) and GL displayed obvious reductions in the ratio of the infarct volume compared with the control group [(33.07 ± 1.74)% in the 10 mg/kg UA group, (27.05 ± 1.13)% in the 20 mg/kg UA group, (27.49 ± 1.87)% in the GL group, and (39.74 ± 2.14)% in the control group] (Figures 1A,C). A significant difference in the infarct volumes was observed between the 10 and 20 mg/kg UA-treated groups.

### Effect of UA on Histological Changes in the Brain of Rats With MCAO/R

**Figure 2:**
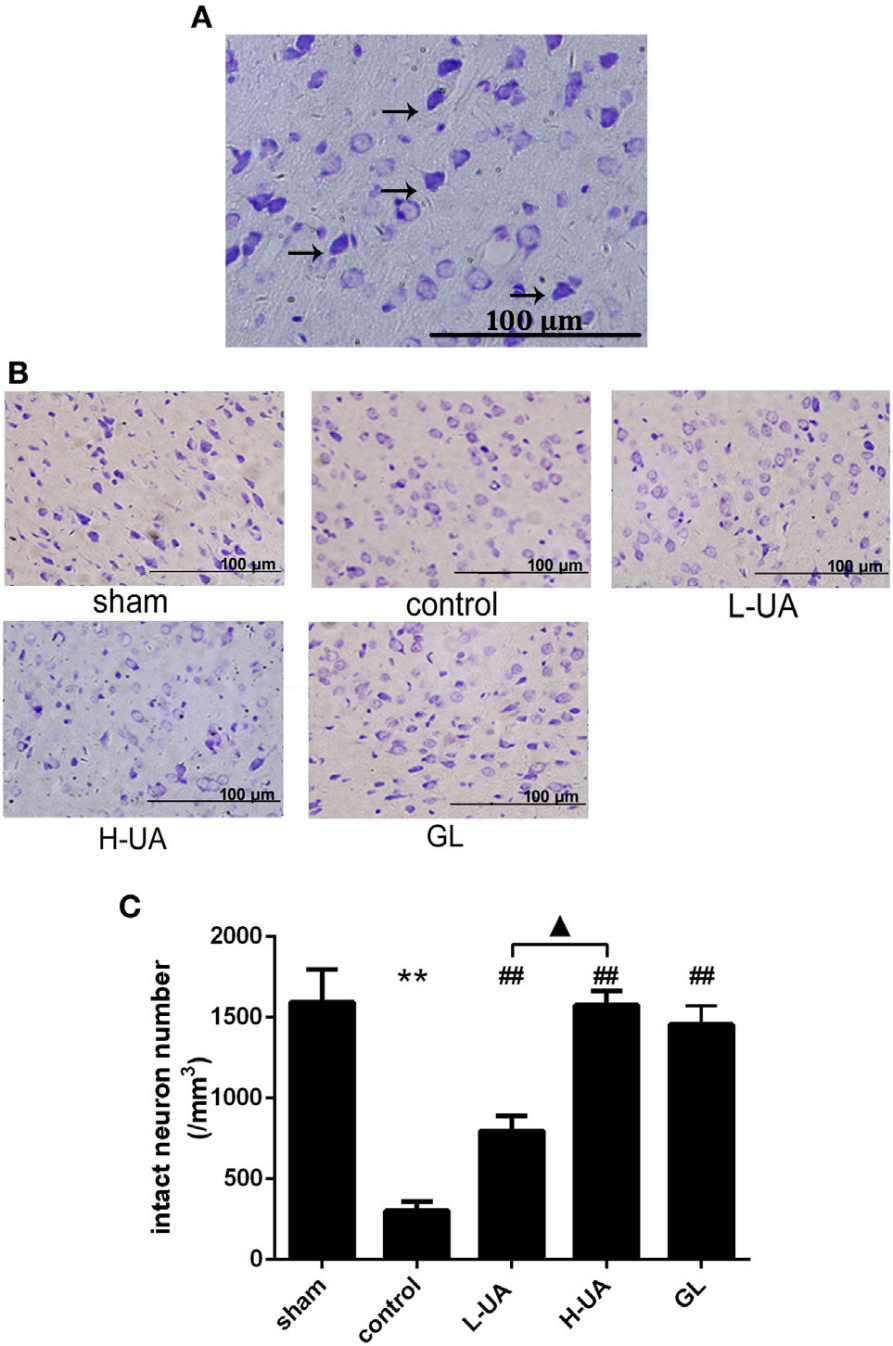
Effect of ursolic acid (UA) on brain damage in rats (values represent the mean ± SE, *n* = 6 per group). **(A)** Nissl staining. Nissl bodies are stained violet. The black arrows indicate the morphology of normal neurons. Normal neurons are intact with clear borders, and the structures are compact with an abundant cytoplasm and Nissl bodies. **(B)** Nissl staining of the cerebral cortical neurons in rats from the different groups. **(C)** The number of intact neurons decreased after MCAO/R, and the number of injured neurons that exhibited cell shrinkage increased (**P < 0.01). UA (10 and 20 mg/kg) and GL increased the number of intact neurons (^##^*P* < 0.01), and the difference between the low-dose UA (L-UA) and high-dose UA (H-UA) groups was significant (^▲^*P* < 0.05) (**P < 0.01: compared with the sham group; ^##^*P* < 0.01: compared with the control group; ^▲^*P* < 0.05: compared with the H-UA group).

Brains were examined histologically by Nissl staining to determine the neuroprotective effects of UA. UA (10 and 20 mg/kg) and GL significantly alleviated the damage in the rat brains. UA and GL increased the number of intact neurons, and the number of injured neurons with cell shrinkage decreased compared with that in the control group (Figure 2). The number of intact neurons increased significantly as the UA concentration increased.

### Effect of UA on Neuronal Apoptosis in the Brain of Rats With MCAO/R

**Figure 3:**
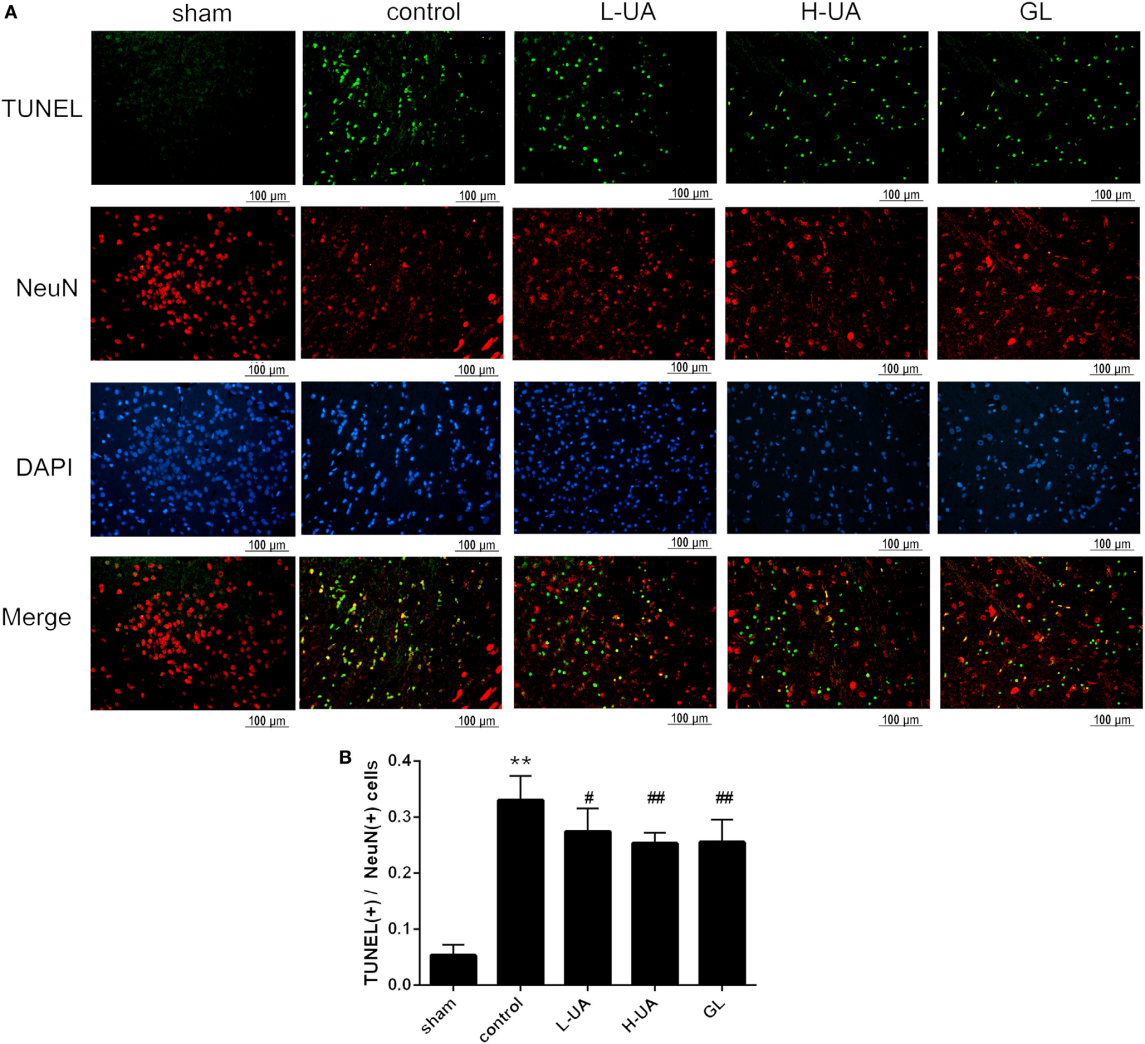
Effect of ursolic acid (UA) on neuronal apoptosis (values represent the mean ± SE, *n* = 6 per group). Double-labeling of transferase-mediated dUTP nick end labeling (TUNEL) and neuronal nuclei (NeuN) in the ischemic region. **(A)** The TUNEL-positive cells were measured by a TUNEL kit. **(B)** A significant reduction in the number of TUNEL-positive neurons was observed in the low-dose UA (L-UA)- (^#^*P* < 0.05), high-dose UA (H-UA)- (^##^*P* < 0.01), and GL-treated groups compared with the control group (^##^*P* < 0.01) (**P < 0.01: compared with the sham group; ^#^*P* < 0.05: compared with the control group; ^##^*P* < 0.01: compared with the control group).

Numerous TUNEL-positive neurons were observed in the ischemic region compared with those in the sham group. A significant reduction of TUNEL-positive neurons was observed in the UA- (10 and 20 mg/kg) and GL-treated groups compared with the control group (Figure 3).

### Effect of UA on Levels of IL-1β, TNF-α, and IL-6 Inflammatory Cytokines in Rats With MCAO/R

**Figure 4:**
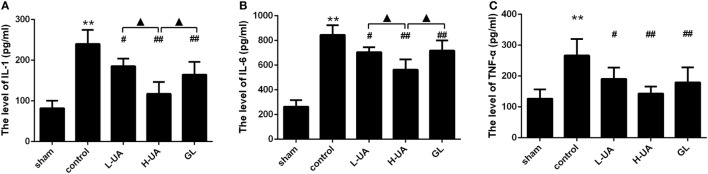
Effect of ursolic acid (UA) on the interleukin (IL)-1β, tumor necrosis factor-alpha (TNF-α), and IL-6 concentrations (values represent the mean ± SE, *n* = 6 per group). Cerebral ischemia and reperfusion increased the IL-1β, TNF-α, and IL-6 levels in the ischemic cortex compared with those in the sham group (**P < 0.01). UA (10 and 20 mg/kg) and GL significantly reduced the IL-1 **(A)**, TNF-α **(B)**, and IL-6 **(C)** concentrations in the ischemic cortex compared with those in the control group (^#^*P* < 0.05; ^##^*P* < 0.01). The 20 mg/kg UA treatment had a stronger effect on IL-1β and IL-6 than the 10 mg/kg UA and GL treatments (^▲^*P* < 0.05) [**P < 0.01: compared with the sham group; ^##^*P* < 0.01: compared with the control group; ^#^*P* < 0.05: compared with the control group; ^▲^*P* < 0.05: compared with the high-dose UA (H-UA) group].

The concentrations of IL-1β, TNF-α, and IL-6 in the ischemic cortex of the control group were significantly higher than those in the sham group after 48 h of reperfusion. The concentrations of IL-1β, TNF-α, and IL-6 were significantly reduced after UA (10 and 20 mg/kg) and GL treatment compared with those in the control group (Figure 4). The 20 mg/kg UA treatment had a stronger effect on IL-1β and IL-6 than the 10 mg/kg UA and GL treatments.

### Effect of UA on HMGB1 and TLR4 in Rats With MCAO/R

**Figure 5:**
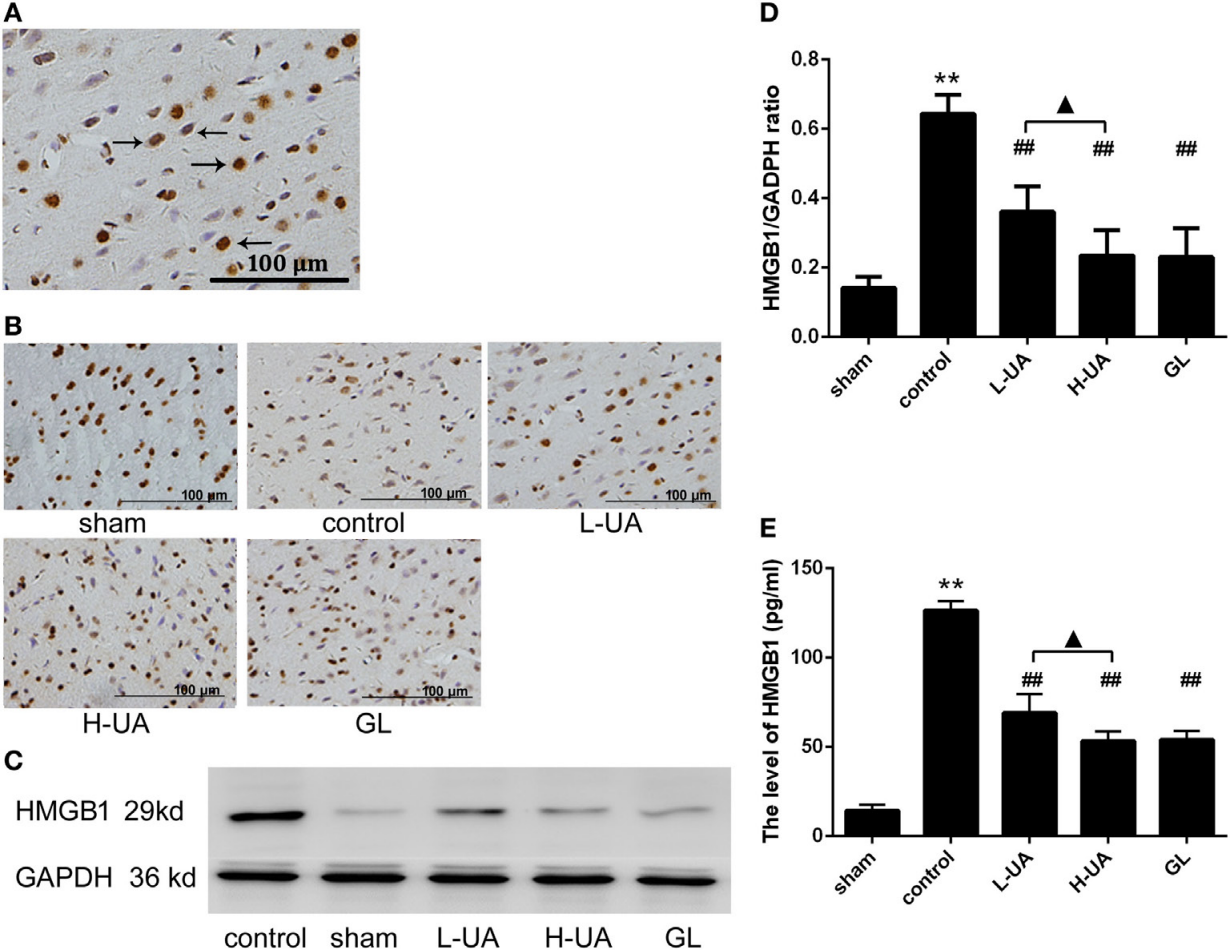
Effect of ursolic acid (UA) on high mobility group box 1 (HMGB1) release (values represent the mean ± SE, *n* = 6 per group). **(A)** Immunohistochemistry staining of nuclear HMGB1 was observed in the cerebral cortex (the black arrows). **(B)** HMGB1 immunochemical staining in the different groups. **(C)** HMGB1 protein expression was also measured by western blotting. **(D)** The target protein bands were densitometrically analyzed and normalized to GAPDH. UA (10 and 20 mg/kg) and GL decreased HMGB1 protein levels (^##^*P* < 0.01). **(E)** The plasma HMGB1 levels were increased compared with those in the sham group (**P < 0.01). UA (10 and 20 mg/kg) and GL significantly reduced the plasma HMGB1 concentrations compared with those in the control group (^##^*P* < 0.01). The difference between the low-dose UA (L-UA) group and the high-dose UA (H-UA) group was significant (^▲^*P* < 0.05) (**P < 0.01: compared with the sham group; ^##^*P* < 0.01: compared with the control group; ^#^*P* < 0.05: compared with the control group; ^▲^*P* < 0.05: compared with the H-UA group).

**Figure 6:**
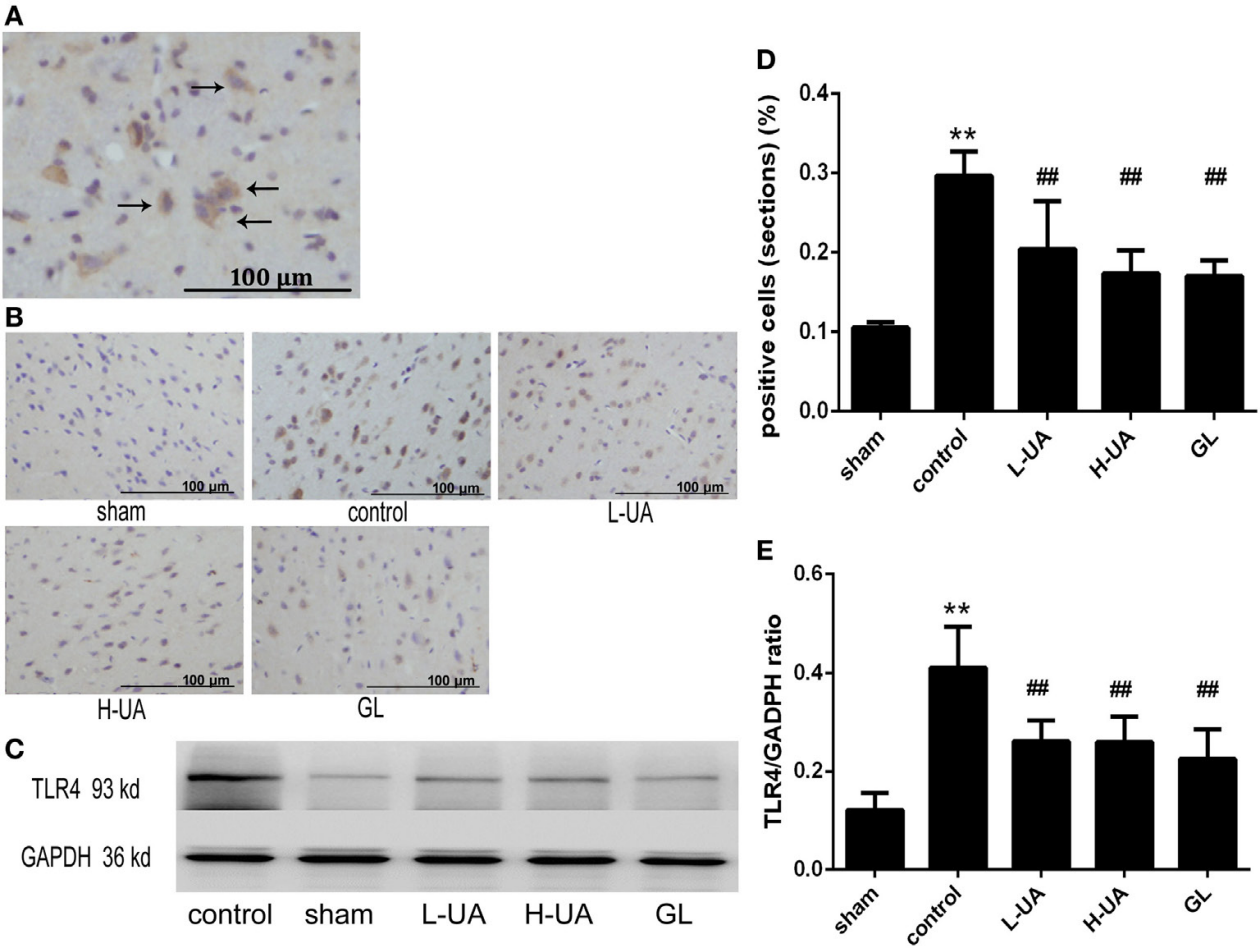
Effect of ursolic acid (UA) on the toll-like receptor 4 (TLR4) levels (values represent the mean ± SE, *n* = 6 per group). **(A)** The expression of the TLR4 protein was measured by immunohistochemical staining. The black arrows indicate TLR4-positive cells expressing TLR4 in the cytoplasm. **(B)** TLR4 immunochemical staining in different groups. **(C)** The expression of the TLR4 protein was also measured by western blotting. **(D)** Quantification of TLR4-positive cells in each group. UA decreased the percentage of TLR4-positive cells (^##^*P* < 0.01). **(E)** The target protein bands were densitometrically analyzed and normalized to GAPDH. UA (10 and 20 mg/kg) and GL decreased TLR4 protein levels (^##^*P* < 0.01) (**P < 0.01: compared with the sham group; ^##^*P* < 0.01: compared with the control group).

Immunohistochemistry and western blotting analyses were performed to confirm HMGB1 and TLR4 expression in the rat brains. Immunohistochemistry staining of nuclear HMGB1 was observed in the cerebral cortex (Figure 5A). However, markedly increased HMGB1 staining was observed in the extracellular space in the control group. The number of nuclear HMGB1-positive cells significantly increased after UA (10 and 20 mg/kg) and GL treatment, and this increase was accompanied by a decrease in extracellular HMGB1 staining (Figure 5B). We also analyzed the brain and plasma HMGB1 levels to measure HMGB1 release. The brain and plasma HMGB1 levels were significantly higher for the control than for the sham group after 48 h of reperfusion. Conversely, the brain and plasma HMGB1 levels were significantly reduced after UA (10 and 20 mg/kg) and GL treatment compared with the levels in the control group (Figure 5). The difference between the 20 mg/kg UA treatment and 10 mg/kg UA treatment was significant. We also observed that UA treatment (10 and 20 mg/kg) and GL treatment decreased the percentage of the percentage of TLR4-positive cells (Figure 6). UA (10 and 20 mg/kg) and GL reduced TLR4immunoreactivity. The semiquantitative immunohistochemical analyses showed the same results as the western blot analyses. Thus, the UA and GL treatments significantly changed the TLR4 protein level (Figure 6).

### Effect of UA on the Activation of Microglia and Astrocytes During MCAO/R in Rats

**Figure 7:**
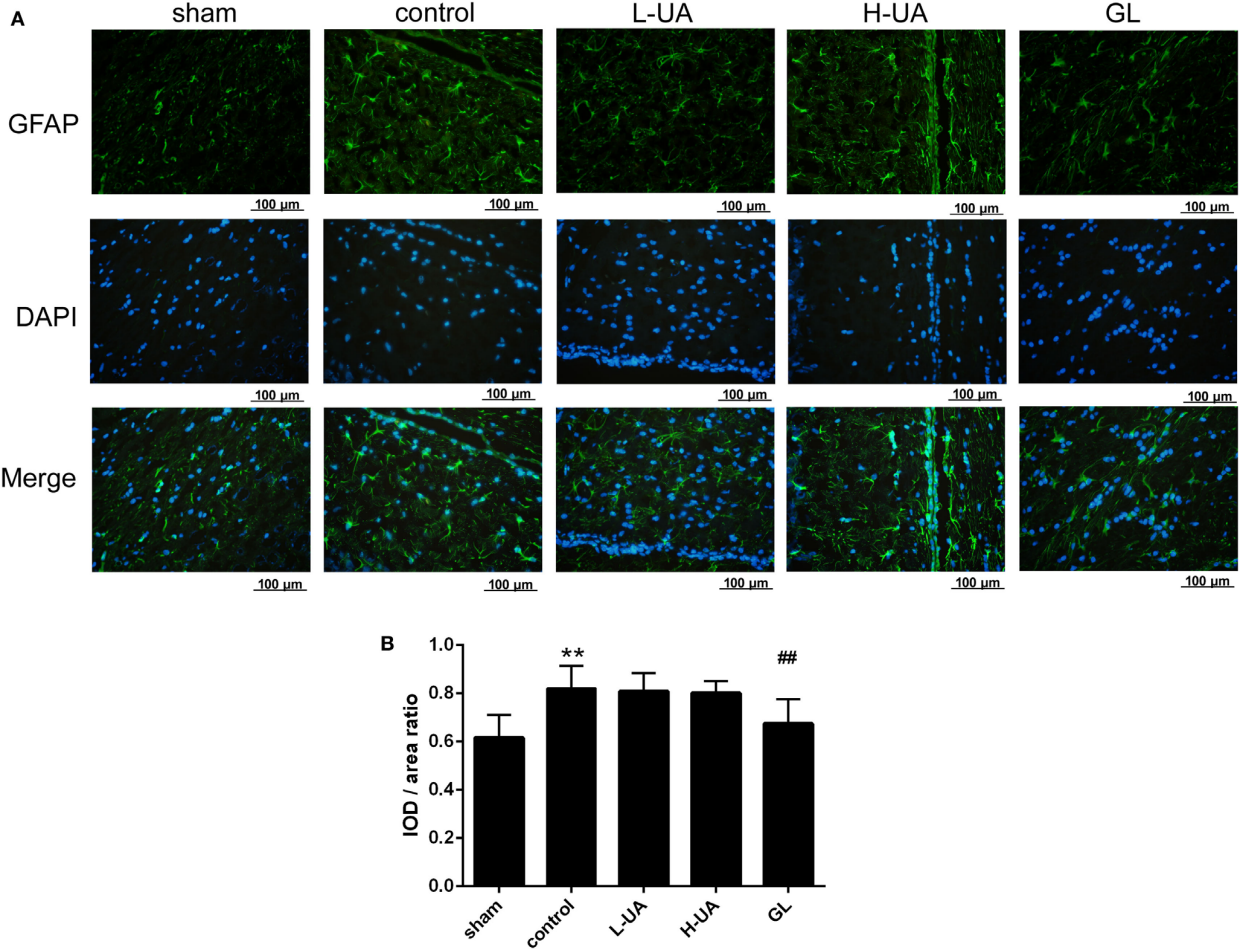
Effect of ursolic acid (UA) on astrocyte activation (values represent the mean ± SE, *n* = 6 per group). Astrocyte activation was confirmed by immunofluorescence staining for GFAP. **(A)** GFAP immunofluorescence staining in the different groups, GFAP (green) and DAPI (blue). **(B)** Quantification of the fluorescence intensity in each group. UA did not change the GFAP fluorescence intensity, but GL decreased the GFAP fluorescence intensity (^##^*P* < 0.01) (**P < 0.01: compared with the sham group; ^##^*P* < 0.01: compared with the control group).

**Figure 8:**
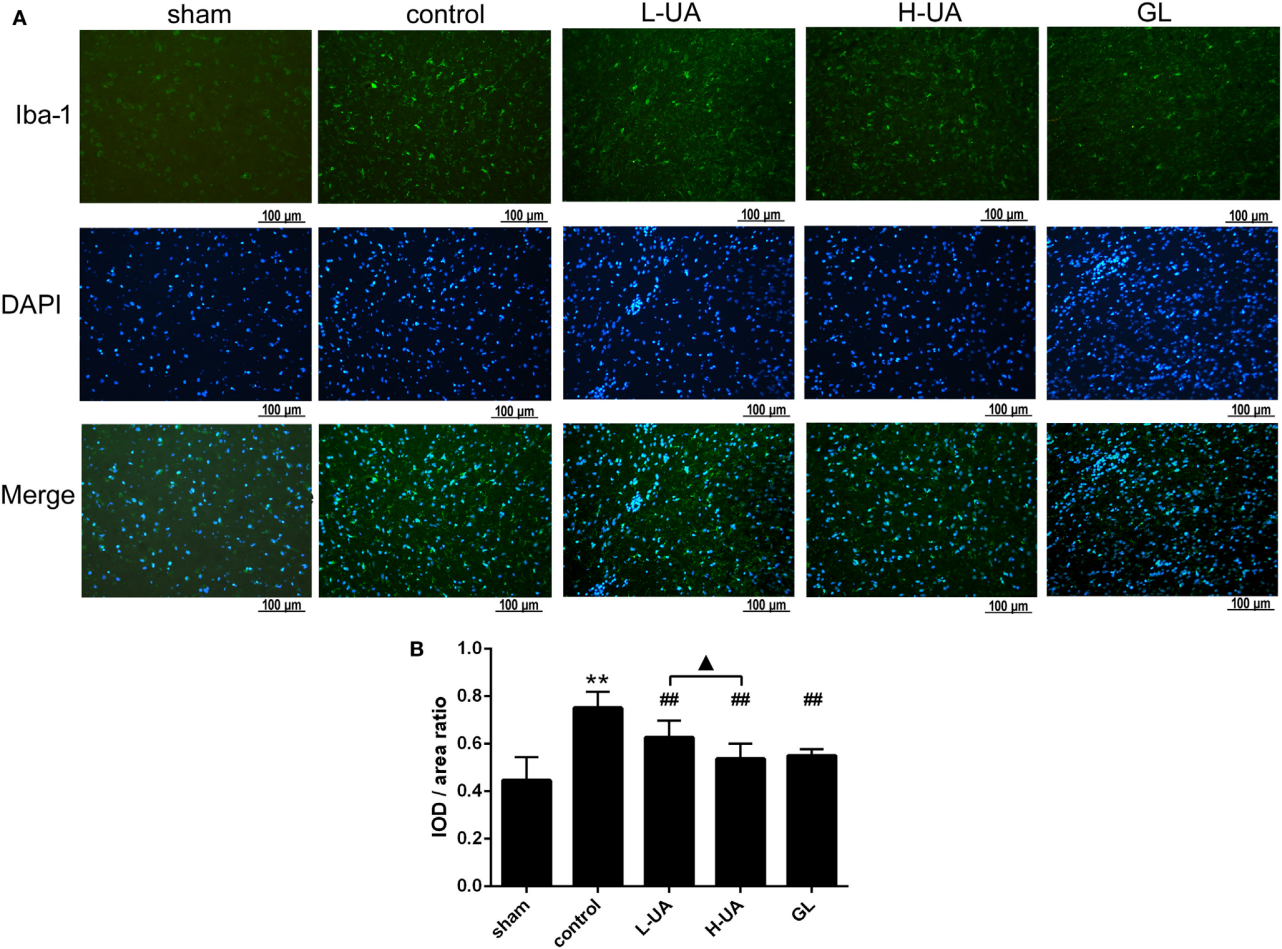
Effect of ursolic acid (UA) on the activation of microglia cells (values represent the mean ± SE, *n* = 6 per group). The activation of microglia was confirmed by immunofluorescence staining for Iba-1. **(A)** Iba-1 immunofluorescence staining in the different groups, Iba-1 (green) and DAPI (blue). **(B)** Quantification of the fluorescence intensity in each group. UA (10 and 20 mg/kg) and GL decreased the fluorescence intensity (^##^*P* < 0.01). The difference between the low-dose UA (L-UA) group and the high-dose UA (H-UA) group was significant (^▲^*P* < 0.05) (**P < 0.01: compared with the sham group; ^##^*P* < 0.01: compared with the control group; ^#^*P* < 0.05: compared with the control group; ^▲^*P* < 0.05: compared with the H-UA group).

Iba-1 and GFAP are specific markers for activated microglia and astrocytes, respectively. Iba-1-positive microglia and GFAP-positive astrocytes were mostly located around the penumbra of the ipsilateral hemisphere in the ischemic brain. MCAO/R significantly increased the Iba-1 and GFAP expression in the ischemic region compared with the sham group. The administration of UA (10 and 20 mg/kg) and GL significantly reduced the Iba-1 expression, whereas UA did not change the GFAP expression (Figures 7 and 8).

### Effect of UA on the NFκB Signaling Pathway in Rats With MCAO/R

**Figure 9:**
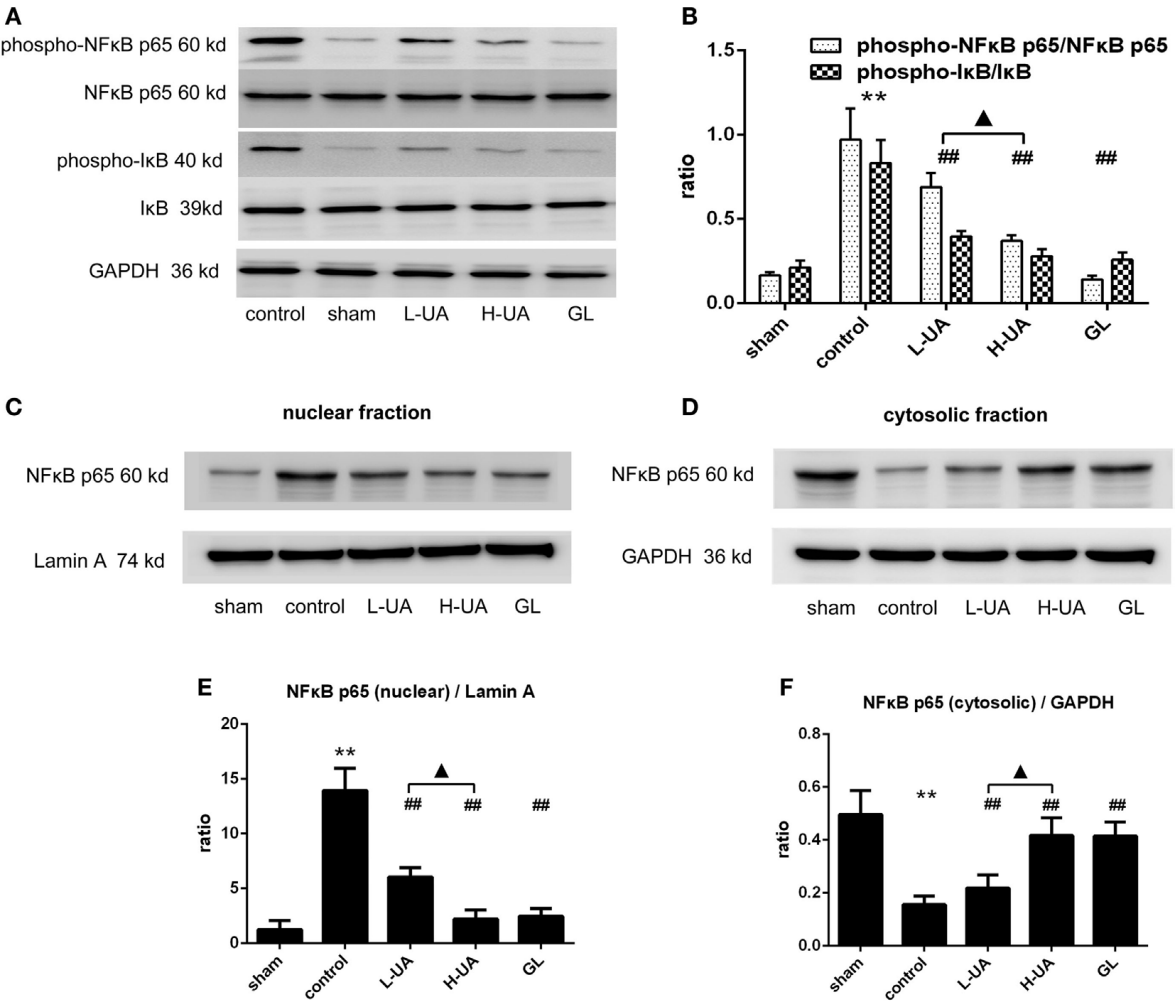
Effect of ursolic acid (UA) on the NFκB signaling pathway (values represent the mean ± SE, *n* = 6 per group). **(A)** Expression patterns of NFκB pathway proteins were measured by western blotting. **(B)** The target protein bands were densitometrically analyzed and normalized to GAPDH. Western blot analysis revealed significantly increased expression of phospho-NFκB p65 and pIκB in the control group compared with the sham group (**P < 0.01). UA (10 and 20 mg/kg) and GL decreased the phospho-NFκB p65 and phospho-IκB expression levels and the translocation of NFκB to the nucleus as determined by immunoblotting of the nuclear and cytosolic fractions **(C–F)** (^##^*P* < 0.01). A significant difference was observed between the low-dose UA (L-UA) group and the high-dose UA (H-UA) group (^▲^*P* < 0.05) (**P < 0.01: compared with the sham group; ^##^*P* < 0.01: compared with the control group; ^#^*P* < 0.05: compared with the control group; ^▲^*P* < 0.05: compared with the H-UA group).

We examined the effect of UA on the NFκB pathway to identify the mechanism by which UA regulates inflammatory cytokines. Western blot analyses revealed significantly higher levels of phospho-IκB and phospho-NFκB p65 in the control group than in the sham group. The UA (10 and 20 mg/kg) and GL treatments significantly decreased the levels of the phospho-IκB and phospho-NFκB p65 proteins. No obvious differences in the levels of total NFκB p65 and total IκB proteins were observed among the experimental groups. We confirmed that UA and GL inhibited NFκB activation, because the cytosolic and nuclear fractions showed decreased NFκB p65 translocation from the cytosol to the nucleus in the UA- and GL-treated rats compared with those in the untreated rats (Figure 9).

## Discussion

Ursolic acid, a pentacyclic terpenoid, exhibits extraordinary neuroprotective properties with anti-inflammatory effects during early brain injury for SAH (31, 32). In our study, UA protected against cerebral ischemia and reperfusion injury by improving neurological deficits and reducing cerebral infarct volumes when administered i.g. at doses of 10 and 20 mg/kg. This finding provides further evidence that UA could be an effective therapeutic agent for cerebral ischemia (17, 33). Our findings provide new insights into the potential effects of UA on brain ischemia.

Cerebral ischemia and reperfusion injury result from the rapid and explosive reoxygenation induced by the inflammatory response, which is tightly associated with inflammatory mediators such as IL-1β, TNF-α, and IL-6. In fact, inflammatory cytokines are involved in aggravating brain infarction both in humans and in experimental stroke models (4, 5, 34–36). Therefore, the regulation of any one of these factors may contribute to reducing ischemic injury. We observed that UA reduces the levels of IL-1β, TNF-α, and IL-6 to modulate ischemic pathology. These cytokine levels were clearly elevated after MCAO/R, and UA significantly inhibited these increases. Moreover, the UA-induced reduction of these cytokines paralleled the reduction of ischemic volume. According to more recent findings, administration of the inhibitor of the IL-1 receptor improved the prognosis in terms of the size of the neurological deficit and the survival rate (37). Mice injected with a neutralizing anti-TNF-α antibody after the induction of stroke exhibited a marked decrease in both infarct volumes and mortality (38). Therefore, UA may be an effective therapy for brain infarction due to its ability to reduce the levels of inflammatory cytokines.

Toll-like receptors are a family of pattern recognition receptors that represent key elements in the initiation and progression of inflammatory cytokine production in response to ischemia and reperfusion injury (39). TLR4 is expressed primarily in microglia and astrocyte in the central nervous system and can be activated by DAMPs (such as HMGB1) to induce downstream signals that lead to cytokine production and thus initiation of an inflammatory response after cerebral ischemia and reperfusion injury (40). In our study, the HMGB1 and TLR4 protein expression levels in the ischemic tissue were reduced and HMGB1 translocation was inhibited after UA treatment. Moreover, HMGB1 and penumbral neuronal apoptosis and death presented the same trends, indicating that UA might have a protective effect against MCAO/reperfusion-mediated HMGB1 release from neural cells, resulting in TLR4 activation. We also observed that UA reduced the expression of Iba1, associated with evidence of microglial activation. Previous studies have shown that TLR4 is a key signaling pathway involved in ischemic penumbral microglial activation, which may be involved in the pathological cerebral conditions by upregulating NFκB (41, 42). In the present study, we examined the levels of NFκB pathway components. The phospho-NFκB p65 and phospho-IκB levels were partially decreased, suggesting that the NFκB signal pathway was inactivated by the inflammatory response after UA treatment. Similar to the results obtained in the present study, TNF-α, IL-6, and IL-1β can be inactivated by a HMGB1 antibody or HMGB1 inhibitor after MCAO/R (43–45). Other reports have shown that NFκB pathway activation is responsible for TLR4-induced target gene expression after hypoxic treatment in microglia (46, 47). Based on these results, the UA-induced reduction in IL-1β, TNF-α, and IL-6 production after MCAO/R may be related to the inhibition of the HMGB1/TLR4/NFκB pathway in microglia. Subsequently, UA attenuated ischemia and reperfusion-induced neuronal apoptosis and death. UA was initially described that modulated potentially of HMGB1/TLR4/NFκB-mediated inflammation and ameliorated cerebral ischemia and reperfusion injury in the present study.

Furthermore, our results showed that UA and GL had similar effects on HMGB1/TLR4/NFκB expression and the reduction of the neurological deficit scores, infarct volume, and apoptosis in penumbral neurons. We used GL, which is the most studied small-molecule inhibitor of HMGB1, as a positive control drug (19, 48). GL has been reported to function as an HMGB1 inhibitor by binding directly to HMGB1 through interactions with the two shallow concave surfaces formed by the two arms of both HMG boxes in a wide number of HMGB1-involved diseases (14, 19, 49, 50). According to our results, the effect of intragastric administration of 20 mg/kg of UA was similar to the effect of intravenous administration of 10 mg/kg of GL on brain ischemia prior to reperfusion. Interestingly, the UA treatment had no effect on astrocytes and a stronger effect on IL-1β and IL-6 during the acute stage of ischemic stroke. Considering that reactive astrocytes in the penumbra of the unaffected area are isolated from the toxic environment of the lesion during the recovery process (51, 52), UA may also benefit patients at risk for ischemic stroke at 2 weeks into recovery stage. Further work is necessary to clarify this point. In addition, clinical studies have utilized liposomes as a drug delivery system to overcome the poor solubility of UA and enhance the bioavailability of this drug (53, 54). These findings above support the possibility and safety of using UA orally to treat cerebral ischemia and reperfusion injury.

However, this study provided only suggestive data. Thus, the mechanism by which UA affected HMGB1 release from the core that led to TLR4-mediated signal transduction was unclear. Further mechanistic studies investigating how UA inhibits of HMGB1/TLR4/NFκB activation are required.

In conclusion, UA attenuated inflammatory cytokine production to protect the brain against cerebral ischemia and reperfusion injury in a rat model possibly through HMGB1/TLR4/NFκB signaling pathway activation. Based on these findings, UA may be useful as a potential effective adjunct to therapy for ischemic brain injury prior to reperfusion.

## Ethics Statement

All experimental protocols involving animals were performed according to the guidelines of the National Institutes of Health Guide for the Care and Use of Laboratory Animals (Publication No. 85-23, revised 1985), the UK Animals Scientific Procedures Act 1986 or the European Communities Council Directive of 24 November 1986 (86/609/EEC). All agreements for animal experiment were approved by the Institutional Animal Care and Use Committee of China medical university and the “Guiding Principles in the Use of Animals in Toxicology,” adopted by the Society of Toxicology in 1989.

## Author Contributions

Y-ZW helped establish the animal model, collect and analyze samples, and write the manuscript. Y-ZW and LL helped perform the Western blotting, immunohistochemistry experiments and review the manuscript. Y-ZW and S-MD helped design the study, establish the animal model, perform data analysis, and write the manuscript. FL helped with drafting the work or revising it critically for important intellectual content. Z-YH contributed to study planning, data analysis and review of the manuscript.

## Conflict of Interest Statement

The authors declare that the research was conducted in the absence of any commercial or financial relationships that could be construed as a potential conflict of interest.
